# On the Use of Microwave Holography to Detect Surface Defects of Rails and Measure the Rail Profile

**DOI:** 10.3390/s19061376

**Published:** 2019-03-19

**Authors:** Andrey Zhuravlev, Vladimir Razevig, Sergey Ivashov, Aleksey Skrebkov, Viktor Alekseev

**Affiliations:** 1 Remote Sensing Laboratory, Bauman Moscow State Technical University, Moscow 105005, Russia; vr@aha.ru (V.R.); sivashov@rslab.ru (S.I.); 2 Russian University of Transport, Moscow 127994, Russia; skrebkov_av@mail.ru (A.S.); alekseevvm@rambler.ru (V.A.)

**Keywords:** microwave holography, rail profile measurement, rail defects, rail inspection, imaging radar, non-contact measurements

## Abstract

The use of microwave holography for detecting rail surface defects is considered in this paper. A brief review of available sources on radar methods for detecting defects on metal surfaces and rails is given. An experimental setup consisting of a two-coordinate electromechanical scanner and a radar with stepped frequency signal in the range from 22.2 to 26.2 GHz is described, with the help of which experimental data were obtained. Fragments of R24 rails with surface defects in their heads were used as the object of study. The radar images of rail defects were obtained by the described method based on back propagation of a wavefront. It is shown that polarization properties of electromagnetic waves can be used to increase the contrast of small-scale surface defects. A method of estimating rail surface profile by radar measurements is given and applied to the experimental data. Comparison of the longitudinal rail head profiles obtained by radar and by direct contact measurements showed that the radar method gives comparable accuracy.

## 1. Introduction

Currently, multichannel ultrasonic systems are used for inspection of rails in which several ultrasonic transducers are placed one after another and move along the rail head. With the help of ultrasound, both external and internal rail defects can be detected. The speed of rail inspection using ultrasonic methods is relatively low because of the need to maintain acoustic coupling between the transducers and the rail head. The acoustic coupling is established by supplying water into the space between the transducers and the rail head. The stability of such a coupling decreases as the speed increases, reducing the reliability of inspection at higher speeds. To measure the geometry of the rail surface, as well as the profile of the rail heads, a non-contact optical triangulation method is used, in which a rail fragment is illuminated by a laser beam and observed with a camera [1]. Using optical methods, the root mean square (RMS) error of 0.34 mm for vertical rail wear and 0.3 mm for horizontal rail wear is reported in [2] for measurements done at the speed of 140 km/h. Optical methods do not require mechanical contact with the examined rail surface, due to which the inspection can be carried out at higher speeds than with ultrasound methods. Disadvantages of optical methods include sensitivity to rail surface contamination as well as the presence of dust, fog, or smoke near the rail tracks.

The emergence of new integrated components in the microwave frequency range on the market, stimulated by advances in mobile telecommunications, application of radars in driver assistance systems, self-driving cars, and microwave personnel screening systems, opens up new possibilities for the use of radars in other fields. One of these potential applications is the detection of surface defects and rail geometry at train operation speeds. Precise measurement of the phase of reflected signals using a quadrature receiver allows for reconstructing the geometry of the rail surface with an accuracy exceeding the accuracy of optical triangulation. It can be estimated that a relative error of 0.05 in phase measurement of a received harmonic signal with the frequency of 30 GHz in a modern harmonic mixer integrated circuit might give an error in radar range estimation equal to a quarter of a millimeter. One advantage of using the radar method compared to optical triangulation is a weaker dependence on the optical properties of the surface being investigated, for example, the presence of surface contamination or rust. Microwave radiation is also less sensitive to the presence of dust or smoke near the rail tracks and the ambient illumination than light. Another advantage of using radar methods could be a simpler implementation of the inspection system, its lower price and operating costs, as well as the possibility of inspecting at ever-increasing train speeds.

There are not many references known to the authors that report the results of using radar to inspect the rail tracks. For example, the use of radar for rail inspection is described in the patent [3]. It describes a diagnostic system consisting of a radar placed on a railway platform with one or two antennas directed toward the rail track. The radar signal processing is described in general terms with a block diagram without presenting any actual radar images. Synthetic aperture radar is given as one of the radar types that can be used in the rail track inspection system.

Radar images of defects in the form of thin cuts in a flat metal plate are given in [4]. The radar data were obtained by mechanical scanning along the plate with an open ended rectangular waveguide oriented parallel to the inspected surface. The side of the waveguide closest to the metal plate with defects did not have a flange, which made it possible to bring the open end of the waveguide closest to the metal surface. The sounding was carried out using frequencies in the range from 26.5 to 40 GHz and from 50 to 75 GHz. Radar images with high spatial resolution, obtained from cuts up to 0.5 mm wide, demonstrate high sensitivity of the method. The sounding geometry used in [4] should be adjusted to provide a standoff distance to be used for practical inspection of rail surface.

Parameters of cracks in a metal surface are measured in [5] using an open-ended waveguide probe oriented perpendicularly to the metal surface and scanning directly on the sample. It is shown that using a sounding signal at the frequency of 20 GHz the parameters of cracks can be measured with submillimeter accuracy. The paper points to the possibility of using polarization properties of electromagnetic waves to increase the sensitivity of the method. The sounding geometry is also near contact and should be adjusted by a standoff distance for inspection of rails. The sensitivity of the described method and the spatial resolution will deteriorate if the antenna moves away from the inspected surface.

The possibility of detecting both external and internal defects of a vibrating rail by measuring the spectra of reflected radar signal is considered in the conference paper [6]. The paper outlines the concept of the method, but does not provide any experimental data from which one could conclude about its capabilities.

These publications indicate that using radar, or antenna probes and appropriate laboratory equipment, one can detect defects on metal surfaces and measure their properties. Publications, in which radar methods are considered within a practically suitable geometry for inspecting rails with experimental results given, are currently not known to the authors. This paper presents such experimental results of rail inspection with practically suitable geometry of sounding that provides a sufficient stand-off distance suitable for high-speed movement of the inspection system.

The remainder of the paper describes an experimental setup, consisting of a mechanical scanner and a radar, with which experimental data were obtained from fragments of rails with artificial defects on their surface. Using the experimental data and the described signal processing algorithm, the capabilities of microwave holography for the inspection of rail surface defects and measuring the longitudinal rail head profile are shown.

## 2. Experimental Setup

**Figure 1:**
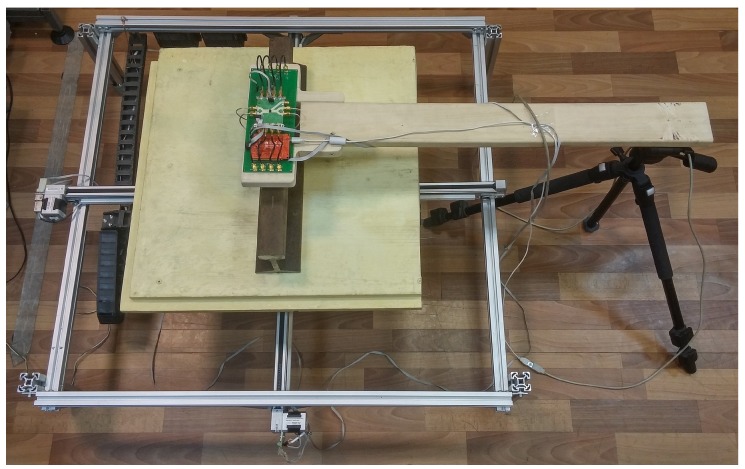
Photo of the experimental setup consisting of two-coordinate mechanical scanner and continuous wave radar with frequency switching.

**Figure 2:**
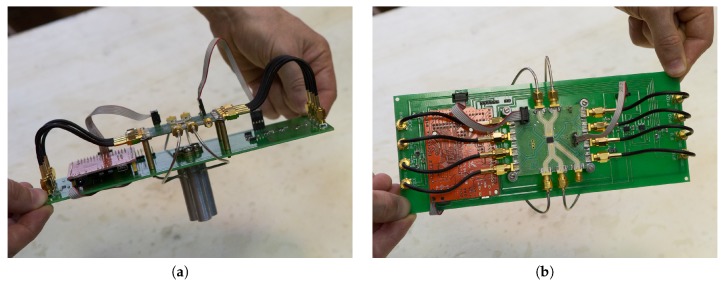
Photo of the radar: (**a**) side view; (**b**) top view.

A photo of the experimental setup is shown in Figure 1, which shows a two-coordinate electromechanical scanner and a radar mounted on one end of a wooden plank, the other end of which is fixed in a tripod. The tripod has an adjustable height, which allows changing the distance from the radar antennas (visible in Figure 2a) to the rail surface.

Photographs of the radar from different views are shown in Figure 2. The radar is built on the basis of Infineon BGT24MTR12 (Neubiberg, Germany), an integrated transceiver, which has one transmit and two receive channels. The frequency of the voltage controlled oscillator is tuned from 22.2 to 26.2 GHz. Circular open ended waveguides were used as antennas because they have wide radiation patterns to increase the size of registered microwave hologram and, hence, improve the radar image resolution. Two receive channels of the radar differ in the direction of linear polarization of radiation. One receive channel is aligned with the direction of polarization of the transmit antenna, and the second receive channel is perpendicular to it. All directions of polarizations of the receive antennas and that of the transmit antenna belong to the same plane.

Resulting from the experiments with the setup, samples of the reflected radar signal above the test object are obtained with programmable sampling intervals at a set of programmable discrete frequencies from the generator frequency tuning range. The obtained samples form an array of two-byte signed integers $Data_{i,j,k,l,m}$, where: *i* is the signal component (0—in-phase, 1—quadrature); *j*—polarization of the received signal (0—parallel to the transmit antenna, 1—perpendicular to the transmit antenna); *k* is the frequency index (0 is the initial frequency, K−1 is the final frequency, where *K* is the total number of frequencies); *l* is the sample row number; and *m* is the sample column number. The resulting data array is written to a file for further processing with the algorithm outlined in the next chapter.

In a radar system suitable for the railway, the mechanical scanning across the rail can be eliminated by applying many electronically switched antennas, so that two-dimensional signal samples are obtained by moving the antenna system along the rail.

## 3. Data Processing

The radar images and the geometry of the rail surface are obtained using the following, based on [7], stages of signal sampling and processing:

1. In a Cartesian coordinate system OXYZ, in which plane XOY is parallel to the rail and axis *Z* points downwards, quadrature samples of the radar signal are recorded in the plane z=0 and stored as an array of complex numbers approximating the function E(x,y,f) on a regular coordinate grid (complex microwave hologram), where *f* is the frequency of the signal, varying from $f_{1}$ to $f_{2}$, and the real and imaginary parts correspond to the in-phase and quadrature components of the signal, respectively. The rail rolling surface (the top surface of the rail head) belongs to the plane $z=z_{a}$.
2. The radar image is calculated according to the back propagation method using the fast Fourier transform (FFT) [8]:
  (1) $$Q\left(x,y,z\right)=FT_{3D}^{-1}\left[FT_{2D}\left[E(x,y,f)\right]\exp\left(-iz_{0}\sqrt{4{\left(\frac{2\pi f}{c}\right)}^{2}-k_{x}^{2}-k_{y}^{2}}\right)\right],$$
  where Q(x,y,z) is the complex reflectivity function, coordinate *z* is measured from the position $z_{0}$; $FT_{2D}$ and $FT_{3D}^{-1}$ denote two-dimensional direct and tree-dimensional inverse Fourier transforms, respectively; k=2πf/c—the wavenumber; *c*—the speed of light; $k_{x}$ and $k_{y}$—coordinate components of the wave vector.
  The absolute value of *Q*, calculated in the plane $z=z_{a}-z_{0}$ by formula (1) is taken as the radar image of the test object.
3. From the set of discrete values $z_{n}$ determined by the inverse three-dimensional FFT in formula (1), ${\hat{z}}_{n}$ is chosen closest to $z_{a}-z_{0}$.
4. At each point $\left(x,y,{\hat{z}}_{n}\right)$, the residual phase Φ(x,y) is calculated by the following expression:
  (2) $$\Phi \left(x,y\right)=\arctan\left(\frac{\mathrm{imag}\left[Q(x,y,{\hat{z}}_{n})\right]}{\mathrm{real}\left[Q(x,y,{\hat{z}}_{n})\right]}\right).$$
5. The distance that corresponds to the height of the rail surface relative to level ${\hat{z}}_{n}$ is calculated according to:
  (3) $$\Delta z\left(x,y\right)=\frac{\mathrm{unwrap}\left[\Phi (x,y)\right]}{2}\frac{c}{2\pi f},$$
  where unwrap() is the function that unwraps the phase by adding a multiple of 2π at the places where the phase jumps due to the function arctan() in Equation (2) reaching a full period.

## 4. Experimental Results

**Figure 3:**
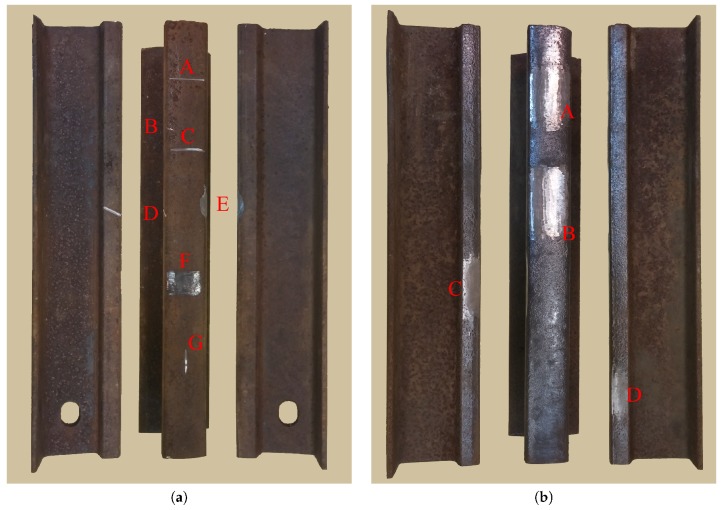
Rail fragments used in experiments: (**a**) rail fragment #1; (**b**) rail fragment #2.

The experiments were carried out with two fragments of R24-type rails (24.96 kg/m) with the length of 50 cm each. The fragments of rails #1 and #2 are shown in Figure 3 with the defects labeled by letters. The dimensions of rail defects such as length, width, and depth are given in Table 1. Measurements of the depth of rail defects were carried out mechanically, using a standard caliper and a metal ruler applied to the rail surface. The defects on rail fragment #1 imitated small-scale defects such as chips and cracks, having at least one dimension much smaller than the radar wavelength, which is equal to 1.25 cm at the frequency of 24 GHz. The defects on rail fragment #2 had dimensions comparable to the wavelength and imitated rail wear or corrugation. From the point of view of electromagnetic wave diffraction on a metal surface, there is no qualitative difference between defects, if their dimensions exceed a wavelength.

**Figure 4:**
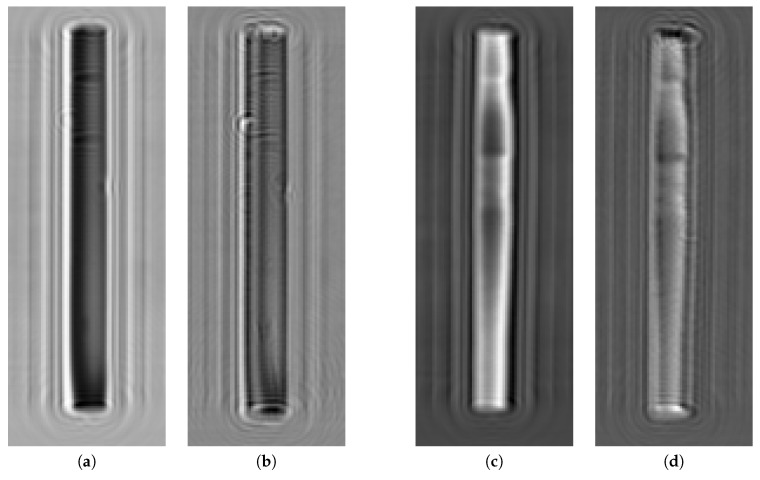
Microwave holograms from rail fragments: (**a**,**b**) rail fragment #1, frequency 24.0 GHz, quadrature components in parallel and perpendicular receive polarizations, respectively; (**c**,**d**) rail fragment #2, frequency 22.2 GHz, quadrature components in parallel and perpendicular receive polarizations, respectively. The incident wave is polarized horizontally and directed along the rail fragments in all cases.

The quadrature components of the radar signal recorded for rail fragments are shown in Figure 4. In this experiment, 21 frequencies, evenly distributed in the interval from 22.2 to 26.6 GHz, were used, so that the frequency step interval was equal to 200 MHz. The sampling interval was equal to 3 mm along both axes. For the rail fragment #1 in Figure 4, the radar signal is shown at the frequency of 24 GHz, while, for the rail fragment #2—at the frequency of 22.2 GHz, as the defects at these frequencies had the highest visual contrast. The measurements were carried out from the distance of 1 cm for the rail fragment #1 and 1.1 cm for the rail fragment #2. The distance to the surface of the rail fragments is about a wavelength and the observable diffraction effects in Figure 4 are relatively weak. The direction of incident radiation was horizontal and directed along the rail fragments in all cases. It can be immediately observed in Figure 4 that the scattered field from the small-scale defects of the rail surface has the highest contrast in the receiving channel of cross polarization, when the reflected signal is received in the polarization perpendicular to the polarization of incident radiation.

**Figure 5:**
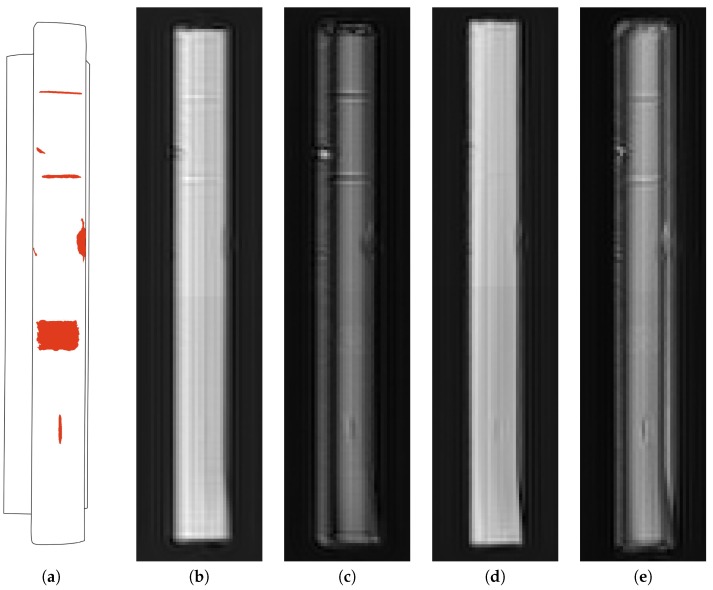
The map of defects and radar images of the rail fragment #1 in various transmit and receive polarizations: (**a**) the map of defects; (**b**) transmit and receive polarizations are along the rail fragment; (**c**) transmit polarization is along, receive polarization is across the rail fragment; (**d**) transmit and receive polarizations are across the rail fragment; (**e**) transmit polarization is across, receive polarization is along the rail fragment.

**Figure 6:**
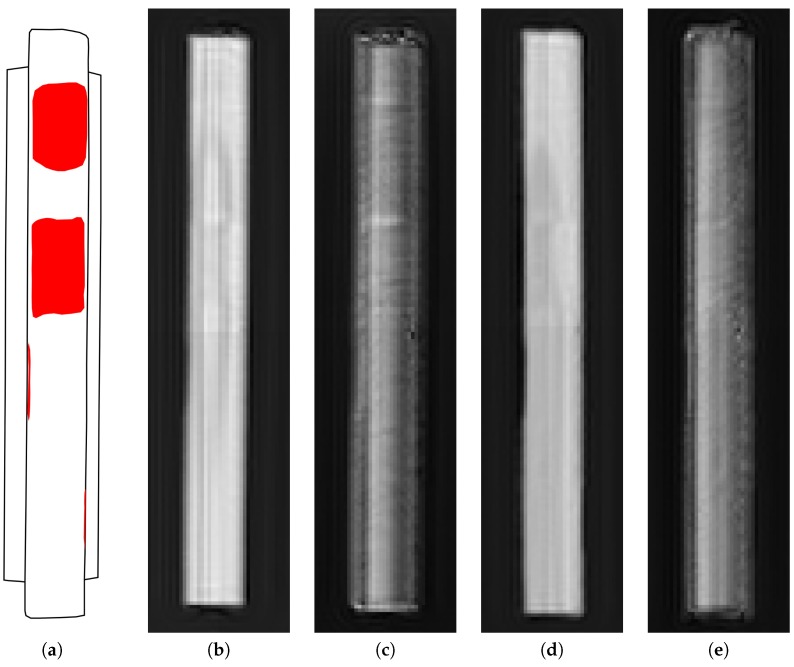
The map of defects and radar images of the rail fragment #2 in various transmit and receive polarizations: (**a**) the map of defects; (**b**) transmit and receive polarizations are along the rail fragment; (**c**) transmit polarization is along, receive polarization is across the rail fragment; (**d**) transmit and receive polarizations are across the rail fragment; (**e**) transmit polarization is across, receive polarization is along the rail fragment.

The results of processing microwave holograms for the rail fragments #1 and #2 are shown in Figure 5 and Figure 6, respectively. Each figure on the left side shows a diagram of a rail fragment, on which the areas of defects are highlighted. The radar images were obtained according to expression (1), in which $z_{a}=1.0$ cm, $z_{0}=0.0$ cm were taken for the rail fragment #1; $z_{a}=1.2$ cm, $z_{0}=0.0$ were taken for the rail fragment #2.

In the radar images shown in Figure 5 and Figure 6, the defects are focused by eliminating the diffraction effects, which occur when the reflected field is detected at a standoff distance from the rail surface. It can be seen in Figure 5 that small defects, which have at least one dimension less than a wavelength, have highest contrast in the cross polarization receive channel. This effect can be used to detect the defects with dimensions smaller than the plan view resolution of the radar system. The intensity of the shown radar images characterizes the absolute value of the reflection coefficient from the rail surface. With this data representation, the phase information contained in the radar signal quadratures is not displayed. The phase information contained in the reflected signal and registered by the radar can be used to calculate the surface geometry of rail fragments.

**Figure 7:**
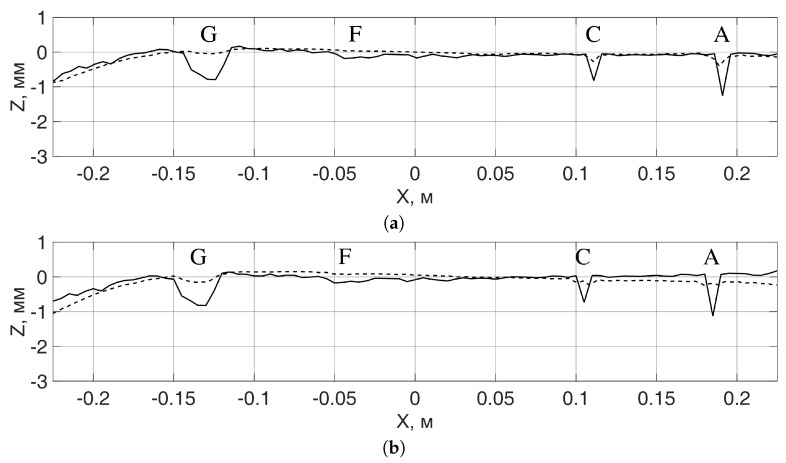
Comparison of contact (solid line) and radar measurements (dashed line) of the surface profile of the rail fragment #1 in the middle line along the rail head: (**a**) both directions of transmit and receive polarizations are along the rail; (**b**) the direction of the transmit polarization is across the rail, the direction of the receive polarization is along the rail.

**Figure 8:**
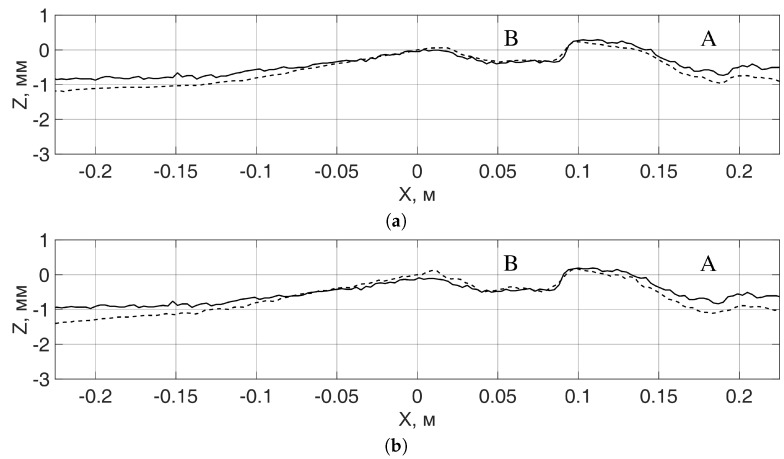
Comparison of contact (solid line) and radar measurements (dashed line) of the surface profile of the rail fragment #2 in the middle line along the rail head: (**a**) both directions of transmit and receive polarizations are along the rail; (**b**) the direction of the transmit polarization is across the rail, the direction of the receive polarization is along the rail.

The longitudinal profile of the rail fragments along the middle line of the rail head was calculated using Equations (1)–(3). The obtained radar profiles were compared with the results of contact measurements, which were done manually using a caliper and a measuring ruler. The ruler was applied to the rail head and the depth gauge of the caliper, which could slide along the ruler, was used to measure the profile at small regular intervals. The corresponding height profiles of the rail heads for the rail fragments #1 and #2 are shown in Figure 7 and Figure 8, respectively. The profile obtained using the radar is shown by a dashed line; the profile obtained using the contact measurements is shown by a solid line. The line on the surface of the rail head, along which the profile was measured, passes through defects A, C, F, and G of the rail fragment #1 in Figure 3, of which defects A, C, and G (from right to left) can be seen on the profile obtained by radar in Figure 7. Defect F, having the coordinate x=−0.05 m on the graph in Figure 7b, is barely distinguished on any of the profiles.

The observable discrepancy between the profiles in the locations of the defects in Figure 7 comes after the fact that the width of the defects is smaller than the plan view resolution of the radar system, which, for the given radar system, is at best equal to a quarter of a wavelength [8], and at the frequency of 24 GHz is around 3 mm. To increase the plan view resolution and reduce the observed discrepancy in the profiles at the places of defects, one can use higher signal frequencies. For the defects larger than a wavelength, the discrepancy between the radar and contact profiles in the areas of defects is substantially smaller, as can be seen in Figure 8.

The discrepancy between the contact and radar profiles in Figure 8, outside the defects, increases smoothly to the edges of the rail fragment. This might result from the error in determining the direction of the middle line of the rail head in radar images, so that the lines of the contact and radar measurements were slightly different.

As it follows from the above figures, microwave holography can be used to detect surface defects and to determine the rail profile with a very high accuracy reaching 0.1 mm as compared with contact measurements. Estimating the physical size of the defect in a plan view radar image, when the size of the defect is much smaller than a wavelength, is not possible directly. For example, the length of a crack, if it is equal to or greater than a wavelength, can be measured directly in the radar image, but the width of the same crack, if it is much smaller than a wavelength, can not. To estimate the dimensions of defects which are smaller than a wavelength indirectly, one can use the observed polarization properties of transmit and receive signals. Such studies can be carried out using the described experimental setup in the future.

## 5. Conclusions

The plan view resolution of the radar images obtained with the described experimental setup depends on the following factors: the distance to the rail surface, the extent of synthetic aperture (depends on directivity pattern of the antenna in use), the sampling interval, and the frequency step of the signal. The best resolution in the plan view (parallel to the scan plane) is approximately equal to a quarter of a wavelength at a stand off distance of a wavelength, if none of the listed above factors is limiting. At the radar frequency of 24 GHz, the plan view resolution is approximately equal to 3 mm. This resolution gives the accuracy with which dimensions of a defect can be estimated directly by radar images. Polarization properties of impinging and scattered radiation can be used to increase the contrast of small, compared to a wavelength, defects on radar images. Quantitative estimation of depolarization factor of scattered radiation can be linked to the physical dimensions of such defects and can also be used for their classification.

The radar range resolution depends on the frequency bandwidth, which in the considered setup is equal to 4 GHz. Such a bandwidth gives a range resolution of around 4 cm, which means that any two point targets can not be resolved if they are at the same spot in the plan view radar image and the distance between them is less than 4 cm. In the experiments shown, the height of the rail profile was determined very accurately, with the precision up to 0.1 mm. The depth of the defects which have at least one dimension smaller than 3 mm (the plan view resolution of the setup) can not be measured with the same accuracy because the small defect and a fragment of the rail are present in the same resolution area in the plan view radar image.

The accuracy of measuring the surface profile of a rail head by the radar method is comparable to the accuracy of optical methods and correlates well with contact measurements. To evaluate the advantage of the radar method for inspecting the condition of the rail surface over optical measurements, it is necessary to compare directly the methods between themselves and the data obtained by accurate contact measurements.

Increasing the plan view resolution of the radar system is possible with higher frequencies. For example, the components designed for automotive radar applications in the frequency range from 77 to 81 GHz can be used for the purpose. This should increase the sensitivity of the radar method to small, compared to a wavelength, defects. The signal sampling interval should be decreased at higher frequencies accordingly.

The experimental data presented in this paper were obtained with a sufficiently small sampling step of 3 mm, which was determined by the proximity of the antenna to the rail surface and the signal frequency. With increasing the distance to the rail surface, the sampling interval can be increased provided that the number of samples is maintained within appropriately larger effective antenna aperture. More experimental data with the radar system for rail inspection can be obtained with the use of an electronically switched antenna system, in which the antennas are distributed in the direction perpendicular to the direction of the rail and the synthetic aperture is obtained by moving the antenna system along the rail on a carriage. Using the experimental setup described in this paper, it is possible to explore various spatial configurations of antenna arrays in order to determine the most optimal, as well as the optimal distance to the rail surface, antenna spacing, and antenna directivity diagram.

## Figures and Tables

**Table 1:** Dimensions of defects on rail fragments #1 and #2.

Rail Fragment Number	Defect Label	Dimensions, mm		
		Length	Width	Depth
#1	A	40	1.0	0.7
	B	12	3.0	2.2
	C	40	2.5	0.7
	D	23	3.0	2.0
	E	30	15.0	0.6
	F	43	25.0	<0.5
	G	30	2.0	1.0
#2	A	60	45.0	0.4
	B	75	45.0	0.5
	C	60	20.0	1.2
	D	45	20.0	0.5
